# Adverse Pathology After Radical Prostatectomy in Low- and Intermediate-Risk Prostate Cancer: A Propensity Score-Matched Analysis of Long-Term Health-Related Quality of Life

**DOI:** 10.3390/diagnostics15151969

**Published:** 2025-08-06

**Authors:** Michael Chaloupka, Alexander Buchner, Marc Kidess, Benedikt Ebner, Yannic Volz, Nikolaos Pyrgidis, Stephan Timo Ledderose, Dirk-André Clevert, Julian Marcon, Philipp Weinhold, Christian G. Stief, Maria Apfelbeck

**Affiliations:** 1 Department of Urology, LMU Klinikum Ludwig-Maximilians-University Munich, Marchioninistr. 15, 81377 Munich, Germany; 2 Institute of Pathology, LMU University, 81377 Munich, Germany; 3 Interdisciplinary Ultrasound Center, Department of Radiology, LMU Klinikum, 81377 Munich, Germany

**Keywords:** adverse pathology, upgrading, radical prostatectomy, HRQOL

## Abstract

**Background and Objective**: Adverse pathology to high-risk prostate cancer (PCa) after radical prostatectomy (upgrading) poses a threat to risk stratification and treatment planning. The impact on sexual function, urinary continence, and health-related quality of life (HRQOL) remains unclear. **Methods**: From 2004 to 2024, 4189 patients with preop low-/intermediate-risk PCa (Gleason score 6 or 7a, PSA ≤ 20 ng/mL) underwent radical prostatectomy at our department and were analyzed. Primary endpoint was HRQOL, erectile function, and urinary continence. Secondary endpoint was rate of salvage therapies and biochemical-free survival. Propensity score matching was performed using “operative time”, “robot-assisted surgery”, “blood loss”, “nerve-sparing surgery”, “age”, and “BMI” to represent comparable surgical approach. Median follow-up was 39 months (Interquartile-range (IQR) 15–60). **Key Findings and Limitations**: Patients who were upgraded to high-risk PCa showed a higher rate of postoperative radiotherapy and androgen-deprivation therapy compared to patients who were not upgraded (21% vs. 7%, *p* < 0.001; 9% vs. 3%, *p* = 0.002). Five-year biochemical recurrence-free survival was 68% in the upgrading group vs. 84% in the no-upgrading group (*p* < 0.001). We saw no difference in patient-reported HRQOL, urinary continence, or erectile function. Multivariable analysis showed that postoperative upgrading was a significant risk for not achieving good overall HRQOL (OR: 0.77, 95% CI: 0.61–0.97, *p* = 0.028) during the follow-up. **Conclusions and Clinical Implications:** Although postoperative upgrading to high-risk PCa leads to worse oncologic outcomes and higher salvage therapy rates, this study indicates that its impact on health-related quality of life is minimal and should not deter a cautious approach to radical prostatectomy.

## 1. Introduction

Prostate cancer is the most common cancer and the third leading cause of cancer-related death in men in Europe [1]. The proportion of patients with low- and intermediate-risk prostate cancer accounts for the majority of new diagnoses [1]. Recently, there has been an increasing trend towards active surveillance of such findings [1], but radical prostatectomy is still a valid treatment option with very good prospects of freedom from metastases and long-term cure [2]. In regards of surveillance as an alternative approach, radical prostatectomy with few side effects is warranted. However, an elevated risk of postoperative upgrading to high-risk prostate cancer may lead the surgeon to omit a bilateral nerve-sparing approach and choose wide-excision strategies instead. This postoperative upgrading is a commonly used scientific tool to evaluate preoperative risk stratification, and multiple studies have identified preoperative risk factors for upgrading [3]. While there is a plethora of data on the oncologic outcomes of each risk group of prostate cancer [4], there is a paucity of data on the impact of postoperative upgrading on long-term quality of life. In the present study, we examine the quality of life of patients who underwent surgery for low- and intermediate-risk prostate cancer but were diagnosed with adverse high-risk prostate cancer postoperatively.

## 2. Patients and Methods

### 2.1. Study Design and Data Evaluation

We retrospectively reviewed our prospectively maintained database of patients who underwent radical prostatectomy at our institution. Between April 2004 and September 2024, 9930 patients underwent radical prostatectomy. Inclusion criteria for the current study were preoperative diagnosis of low- or intermediate-risk prostate cancer according to risk stratification by d’Amico et al. and current guidelines: Gleason score 6 or 7a and PSA ≤ 20 ng/mL [1,5]. The optional definition of low- or intermediate-risk prostate cancer in terms of local confinement as assessed by digital rectal examination, multiparametric resonance imaging, and randomized biopsy of the prostate is not documented in our database and therefore not included in the analysis. Likewise, intermediate-risk patients are not separated into favorable and unfavorable intermediate-risk. Patients who received neoadjuvant treatment were excluded from the study. This left 4189 patients (42%) eligible for analysis. Patients were then divided into two groups according to either adverse pathology (Gleason Score > 7a) to high-risk prostate cancer (upgrading, n = 727/4189 (17%)) or concordance with preoperative diagnosis (no upgrading, n = 3462/4189 (83%)). We performed a propensity score-matched analysis including the variables “operative time”, “robot-assisted radical prostatectomy”, “blood loss”, and “nerve-sparing surgery” to represent comparable surgical care. We also included the variables “age” and “body mass index (BMI)” in the propensity score matching, as these variables have been shown to be significant confounders of health-related quality of life [6,7].

Patient-reported outcome measures were collected by mail immediately before radical prostatectomy and at 3 months, 1 year, 3 years, and 5 years postoperatively. Health-related quality of life (HRQOL) was assessed using the EORTC QLQ-C30 questionnaire. In accordance with current EORTC guidelines, overall HRQOL was determined by the Global Health Status (GHS) domain, with a score of 70 or higher indicating good overall HRQOL [8]. Erectile function was assessed using the International Index of Erectile Function (IIEF-5) questionnaire, with a maximum score of 25 and a score of 18 or higher indicating satisfactory function [9]. Urinary continence was measured using the validated short form of the International Consultation on Incontinence Questionnaire (ICIQ-SF); this tool rates severity from 0 to 21, with higher scores reflecting more severe incontinence [10]. Continence recovery was defined as the use of up to one security pad within a 24 h period. Biochemical recurrence was defined as two consecutive PSA levels of at least 0.2 ng/mL. Biochemical recurrence-free (BCR) survival was compared between patients with and without upgrading using Kaplan–Meier analysis and logrank test.

### 2.2. Statistical Analysis

Testing for normal distribution of variables was performed with Shapiro–Wilk test. Continuous variables are reported as medians with interquartile ranges (IQR), while categorical variables are presented as absolute counts with corresponding percentages. The Mann–Whitney U test was used for comparing continuous variables, and the chi-squared (χ^2^) test was applied for categorical variables. We conducted a multivariable logistic regression analysis to evaluate the impact of upgrading on good overall HRQOL, satisfactory erectile function and continence recovery. For each outcome, odds ratios (ORs) along with 95% confidence intervals (CIs) were estimated. Statistical analyses were performed using R (version 3.6.3), Prism 6 (GraphPad Software, San Diego, CA, USA), and MedCalc version 23 (MedCalc, Ostend, Belgium). Two-sided *p*-values < 0.05 were considered statistically significant. This study received approval from the local ethics committee (#20-1022, date of approval: 5 June 2021).

## 3. Results

### 3.1. Perioperative Patient Characteristics and Oncological Outcomes

**Figure 1:**
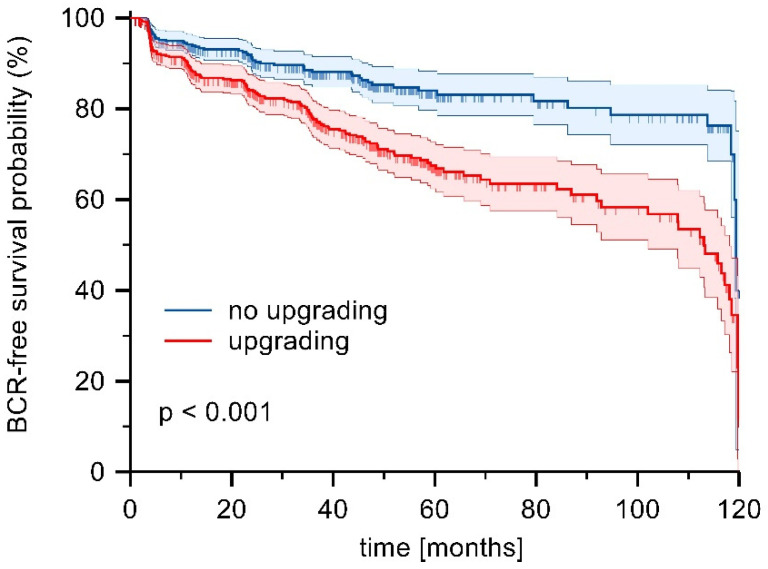
Biochemical recurrence (BCR)-free survival for patients upgraded after radical prostatectomy compared to patients not upgraded after radical prostatectomy. Logrank test: *p* < 0.001.

Detailed patient characteristics of the unmatched patient cohort are summarized in Table 1. Briefly, median patient age, PSA, and Gleason score were significantly different between groups (*p* < 0.001 for each). We then created a propensity score matched cohort as described above (Table 1). The matched cohorts showed no significant differences in age, BMI, operative time, rate of robotic-assisted radical prostatectomy, blood loss, and nerve-sparing surgery to represent a comparable surgical approach and to exclude possible confounders of health-related quality of life. Median follow-up was 39 months (IQR: 15–60). Follow-up was available for 80% of upgraded patients and 77% of patients who were not upgraded postoperatively. PSA was significantly higher in patients who were upgraded postoperatively compared to patients who were not upgraded (8.0 ng/mL vs. 6.9 ng/mL, *p* < 0.001). In addition, preoperative ratio of Gleason 7a prostate cancer was significantly higher in patients who were upgraded compared to patients who were not upgraded after radical prostatectomy (72% vs. 48%, *p* < 0.001). The majority of patients who were upgraded postoperatively were ultimately diagnosed with Gleason 7b prostate cancer (72%). Patients who were upgraded post-operatively had a significantly higher amount of extracapsular extension compared to patients who were not upgraded post-operatively (52% vs. 18%, *p* < 0.001). Likewise, patients who were upgraded postoperatively underwent significantly more often postoperative radiation and androgen-deprivation therapy during follow-up (21% vs. 7%, *p* < 0.001; 9% vs. 3%, *p* = 0.002). Furthermore, biochemical recurrence-free survival after five years was 68% in the upgrading group compared to 84% in the no upgrading group (*p* < 0.001) (Figure 1).

### 3.2. Functional Outcomes and Health-Related Quality of Life

**Figure 2:**
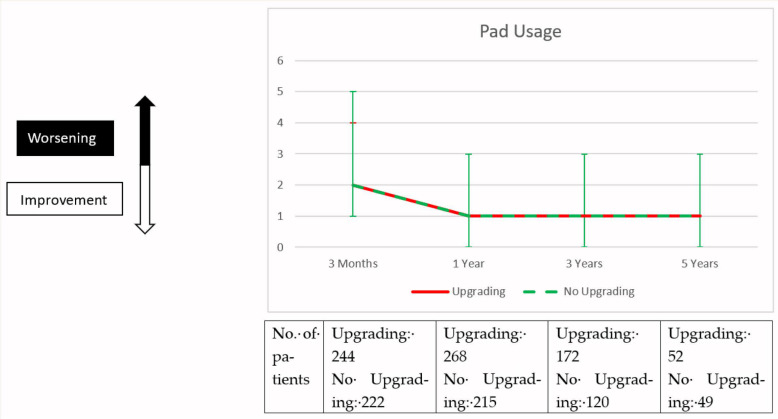

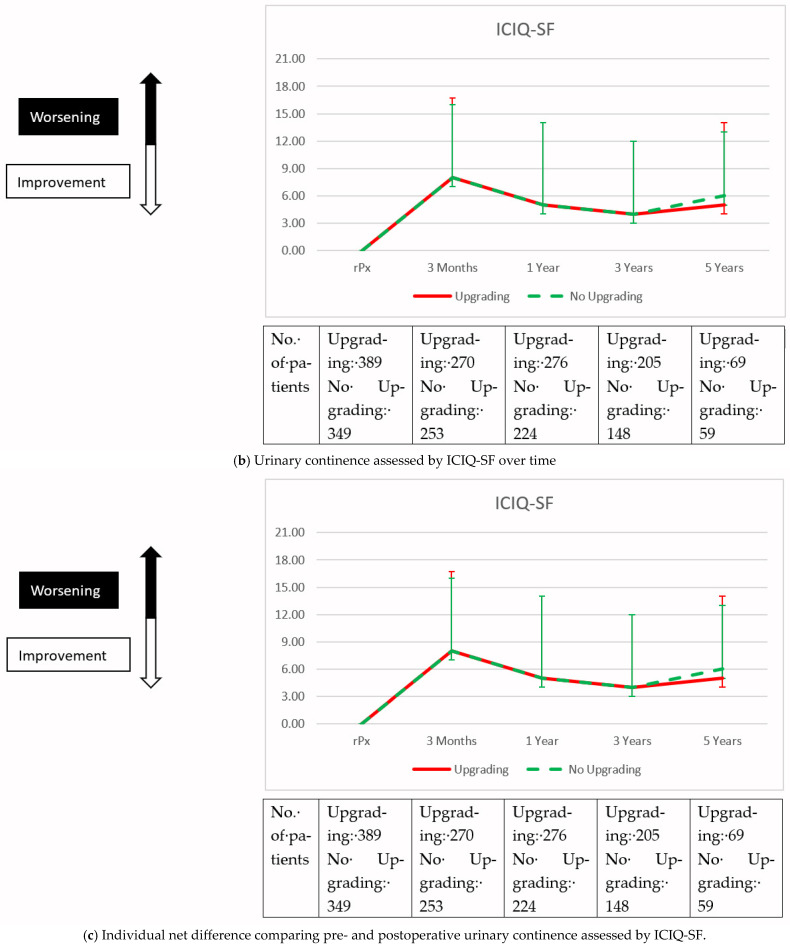

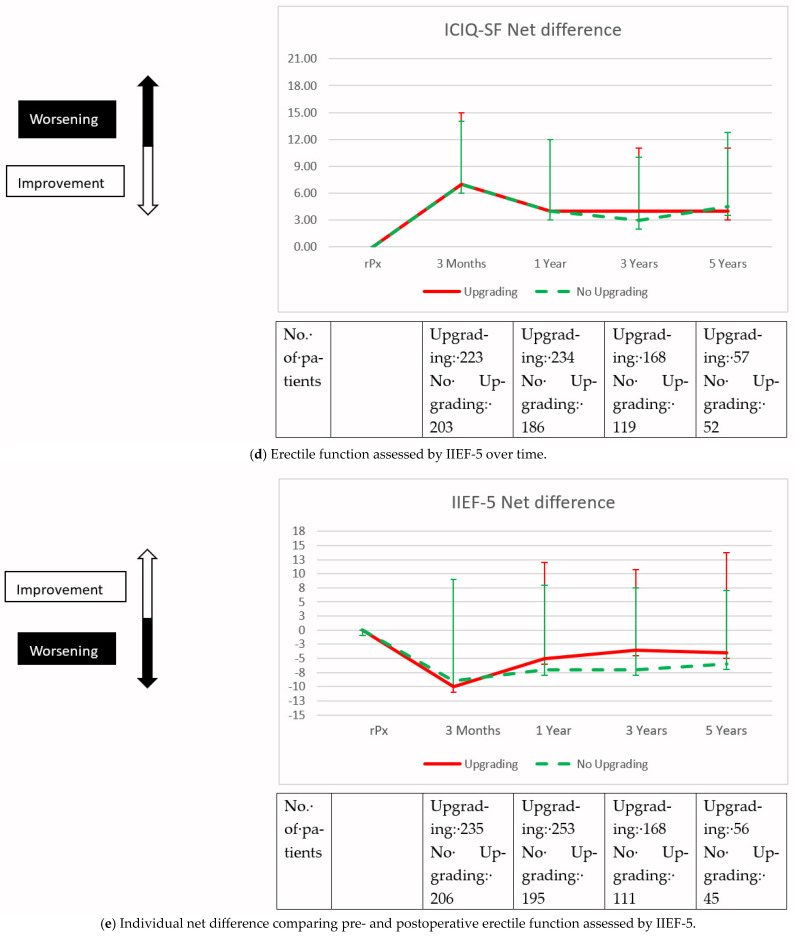

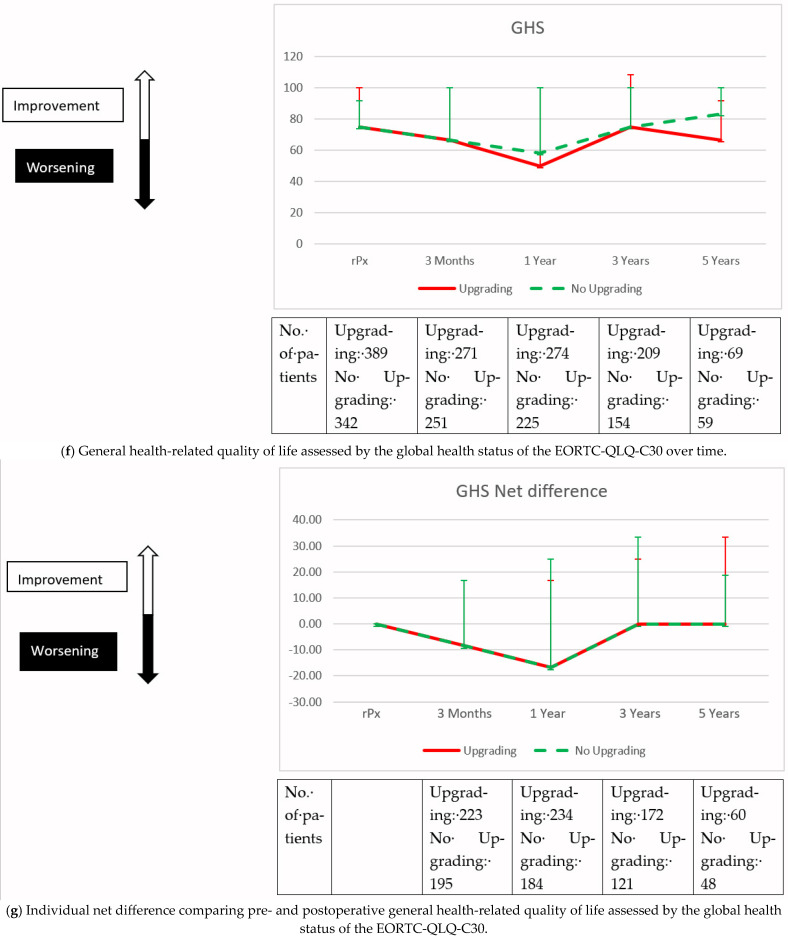
Health-related quality of life outcomes of propensity score-matched patients upgraded after radical prostatectomy compared to patients not upgraded after radical prostatectomy. Display of mean and interquartile range of the cohorts and individual net difference to baseline before radical prostatectomy. rPx: radical prostatectomy; ICIQ-SF: International Consultation of Incontinence Questionnaire–Short-Form; IIEF-5: International Index of Erectile Function; GHS: Global Health Scale; EORTC-QLQ-C30: European Organization for Research and Treatment of Cancer quality of life questionnaire.

Preoperative and postoperative functional outcomes and overall health-related quality of life are displayed in Figure 2. We compared the pooled mean value of the cohorts, as well as the net difference from the individual’s baseline before radical prostatectomy. Briefly, when comparing upgraded and non-upgraded patients, we found no significant difference in urinary continence, erectile function and good overall HRQOL as assessed by ICIQ-SF, IIEF-5, GHS Domain of the EORTC-QLQ-C30 questionnaires and daily pad use, before and after radical prostatectomy. This was evident when comparing the cohorts at each time point of follow-up, as well as when comparing the individual net difference from the preoperative baseline. The only difference was in the comparison of postoperative erectile function one year after radical prostatectomy, where the upgraded cohort had worse erectile function compared to the non-upgraded cohort after radical prostatectomy (mean IEEF-5 score: 5 vs. 6, *p* = 0.008). However, this disadvantage could not be demonstrated in the subsequent follow-up. In multivariable logistic regression analysis, we saw that the factor of postoperative upgrading was a significant risk factor for not achieving good overall HRQOL (GHS ≥ 70) during follow-up (OR: 0.77, 95% CI: 0.61–0.97, *p* = 0.028). However, we did not see a significant association between postoperative upgrading and satisfactory erectile function (IIEF-5 score ≥ 18) and recovery of urinary continence (up to 1 pad per day) during follow-up (Table 2).

## 4. Discussion

Adverse pathology after radical prostatectomy is a common phenomenon and can undermine preoperative risk stratification and treatment planning. When in doubt, the risk of postoperative upgrading may lead to a more extensive radical prostatectomy and, consequently, to a reduction in postoperative quality of life. The oncologic outcome of postoperative reclassification has been studied extensively, but little is known about the impact of upgrading on postoperative HRQOL. In the present analysis, we examined the long-term impact of postoperative upgrading on HRQOL in a cohort of patients with the most severe consequences of upgrading: low- and intermediate-risk prostate cancer to high-risk prostate cancer. In our cohort, we observed a significant difference in oncologic outcome and subsequent salvage therapies between patients who underwent postoperative upgrading and those who did not. We saw no significant difference in the comparison of long-term outcomes for erectile function, urinary incontinence, and general HRQOL. However, in the multivariable analyses, the fact of postoperative upgrading was identified as an independent risk factor for achieving good overall HRQOL. Consequently, it can be concluded that the impact of postoperative upgrading of low- and intermediate-risk prostate cancer to high-risk prostate cancer on HRQOL is low and should not affect a careful approach to radical prostatectomy.

Radical prostatectomy of high-risk prostate cancer has a high chance of recurrence and the need for salvage therapies. Untreated, high-risk prostate cancer has a 10-year cancer-specific mortality rate of approximately 30% [11]. To date, no optimal treatment regimen has been recommended by guidelines, and multimodal treatment, including radical prostatectomy, is likely necessary [1]. Potential salvage options include external beam radiation therapy in combination with androgen-deprivation therapy. Irrespective of its timing, these therapies proved to increase prostate cancer treatment-specific side effects in terms of genito-urinary toxicity and sexual function, mood and cognitive function [12,13,14,15]. This is also reflected in our long-term outcomes data. Patients who were upgraded to high-risk prostate cancer after radical prostatectomy had a higher risk of biochemical recurrence after five years compared to patients who were not upgraded to high-risk prostate cancer (48% vs. 68%; *p* < 0.001). In addition, patients who were upgraded to high-risk prostate cancer after radical prostatectomy received significantly more often postoperative radiation therapy and androgen deprivation therapy compared to patients who were not upgraded to high-risk prostate cancer (21% vs. 7%, *p* < 0.001; 9% vs. 3%, *p* = 0.002). Both therapies can have a negative impact on quality of life. This is a potential confounder in our multivariable analysis, showing that postoperative upgrading is a significant risk factor for achieving good overall HRQOL (OR: 0.77, 95% CI: 0.61–0.97, *p* = 0.028).

To maintain erectile function and urinary continence, preservation of the neurovascular bundles with parasympathetic nerve branches of the pelvic plexus is critical during radical prostatectomy [16]. However, locally advanced high-risk tumors may require a wide resection that damages these delicate structures. Earlier guidelines considered the clinical suspicion of extracapsular extension or more than one positive Gleason ≥7a ipsilateral biopsy to be a contraindication to a nerve-sparring approach in order to reduce the risk of positive surgical margins [17]. Studies have shown a correlation between extracapsular growth, nerve preservation and positive postoperative surgical margins [18]. In fact, the rate of nerve-sparing radical prostatectomy is significantly lower in our cohort of patients with postoperative upgrading than in the cohort of patients without postoperative upgrading (82% vs. 92%, *p* < 0.001). A possible explanation is the significantly higher incidence of extracapsular extension in patients with postoperative upgrading compared to patients without postoperative upgrading (Unmatched cohort: 52%. vs. 15%, *p* < 0.001; Matched cohort: 52% vs. 18%, *p* < 0.001). Despite the high rate of nerve-sparing procedures in our cohort, erectile function deteriorated after radical prostatectomy in no-upgrading and upgrading cohort, with a median IIEF-5 decline of 4 and 6 points, respectively. Although patients experienced recovery during follow-up, the median IIEF-5 did not return to baseline. This is consistent with other studies evaluating erectile function after nerve-sparing prostatectomy: Michl et al. reported a median postoperative IIEF-5 drop of 7 points in 411 patients, with outcomes significantly better following unilateral or bilateral nerve preservation versus no nerve preservation [19]. Overall, the prevalence of potency in our cohort aligns with the wide range of 26–63% observed in meta-analyses [20]. The advanced age of our study cohort (median age 67 years) may represent a potential confounder, as studies demonstrate an increased prevalence of erectile dysfunction with advancing age [21]. Notably, the baseline median IIEF-5 score at the time of surgery was 15, reflecting mild to moderate preexisting erectile dysfunction. A positive surgical margin was significantly more common in patients with postoperative upgrading compared to patients without postoperative upgrading (Unmatched cohort: 31%. vs. 15%, *p* < 0.001; Matched cohort: 30% vs. 15%, *p* < 0.001). Positive surgical margins are associated with a higher risk biochemical-recurrence. However, their impact on prostate cancer-specific survival is highly variable [18]. In view of the high likelihood of multimodal therapy for high-risk carcinoma and the variable influence of positive resection margins on the oncologic outcome, patients should be informed about the necessary extent of the resection at the time of radical prostatectomy and its impact on HRQOL. Studies show that nerve-sparing surgery is feasible in preoperatively diagnosed high-risk prostate cancer patients. Furthermore, in a large retrospective multicenter study of 4351 patients with high-risk prostate cancer, Preisser et al. showed that nerve-sparing radical prostatectomy was not associated with worse oncological outcomes compared to patients without nerve-sparing radical prostatectomy [22]. Our data support this prioritization of nerve-sparing. Assuming the same surgical approach, we saw no difference in the long-term course of erectile function between patients with low- and intermediate-risk prostate cancer and postoperative high-risk prostate cancer. After propensity score-matching (“operative time”, “robot-assisted radical prostatectomy”, “blood loss”, and “nerve-sparing surgery”) to ensure comparable surgical care, long-term follow-up shows that the functional outcome in terms of erectile function does not differ significantly between the two cohorts. It remains unclear whether this lack of difference would also exist if the patients with postoperative upgrading to high-risk prostate cancer had been treated with wider resection to avoid positive surgical margins. Several studies have investigated the risk of postoperative upgrading and created a nomogram to identify such patients preoperatively [23,24]. However, the purpose of such nomograms remains questionable. Our data show that these nomograms should influence the decision for or against surveillance, but not the approach to surgery.

There are several limitations to our study. First, preoperative multiparametric MRI (mpMRI) of the prostate is not documented in our prospective database and was therefore not included in the analysis. Furthermore, patients with mpMRI-targeted biopsy are not documented and were not analyzed separately. Meta-analyses show that the combination of mpMRI-targeted biopsy and randomized biopsy detects more patients with high-risk prostate cancer preoperatively [25]. This might have altered preoperative risk stratification of patients who underwent mpMRI-targeted biopsy in our study cohort. However, previous studies about patients who underwent mpMRI-targeted biopsy at our institution did not show a different risk of postoperative upgrading compared to patients who did not undergo mpMRI-targeted biopsy [26,27]. Second, mpMRI is a valuable tool to assess local confinement and surgical planning and, if available, may have influenced the surgical approach in our study cohort. However, studies show that the positive predictive value of mpMRI in the prediction of extracapsular extension is particularly low in patients with low- and intermediate-risk prostate cancer and should therefore be interpreted with caution [28]. Third, in our study design, we defined postoperative upgrading based solely on the differentiation of prostate cancer in accordance with current guidelines [1]. However, the presence of postoperative extracapsular extension can also be classified as high-risk prostate cancer regardless of its differentiation. Accordingly, we would have to assign all patients with postoperative extracapsular extension to the upgrading cohort. However, current guidelines consider the combination of low differentiation and extracapsular extension as a high-risk factor [1]. Furthermore, studies comparing adjuvant versus salvage radiotherapy after radical prostatectomy also show that extracapsular extension alone is not a compelling risk factor and can be initially observed or treated with salvage radiotherapy [12,13,14]. Next, because of the lack of information on clinically assessed local confinement in our database, we could not separate intermediate-risk patients into favorable and unfavorable risk as recommended by current guidelines [1]. Furthermore, we are unable to differentiate between unilateral and bilateral nerve-sparing, despite evidence that this distinction significantly affects postoperative erectile function [19]. The final limitation is inherent to the retrospective, single-center design of our study. However, to our knowledge, we report on one of the largest single-center cohort studies with adequate follow-up on this matter.

## 5. Conclusions

In summary, we investigated the effect of postoperative upgrading of low- and intermediate-risk prostate cancer to high-risk prostate cancer after radical prostatectomy on health-related quality of life after long-term follow-up. Despite worse oncologic outcomes and higher salvage therapy rates, the results of this study show that the impact of postoperative upgrading to high-risk prostate cancer on health-related quality of life is low and should not influence a cautious approach to radical prostatectomy.

## 6. Take Home Message

Our study demonstrates that while postoperative upgrading from low- and intermediate- to high-risk prostate cancer leads to poorer oncologic outcomes and higher salvage therapy rates, it does not necessarily have a negative impact on health-related quality of life.

## Figures and Tables

**Table 1:** Patient characteristics. Continuous values are presented as median and inter-quartile-range (IQR); categorical values are given as number (n; %). BMI: body mass index; PSA: prostate specific antigen; PreOP: preoperative; PostOP: postoperative; RP: radical prostatectomy; ADT: Androgen-deprivation therapy. ^(a)^ Propensity score-matching included the following variables: Age, BMI, Robot-assisted RP, Nerve-sparing RP, Blood loss, OP duration.

**Unmatched Cohort**			
	**No Upgrading**	**Upgrading**	***p***
No. of patients	3462	727	
Age, years [median, IQR]	65 (59–70)	67 (62–72)	<0.001
BMI, kg/m^2^ [median, IQR]	26 (24–28)	26 (25–29)	0.02
PSA preop., ng/mL [median, IQR]	6.7 (5.0–9.3)	8.0 (5.8–12)	<0.001
Gleason Score preOP [n (%)]			
6	1813/3462 (52%)	207/727 (28%)	<0.001
7a	1649/3462 (48%)	520/727 (72%)	
Gleason Score postOP [n (%)]			
6	1238/3462 (36%)		
7a	2224/3462 (64%)		
7b		525/727 (72%)	
8		146/727 (20%)	
9		55/727 (8%)	
10		1/727 (0.1%)	
Extracapsular extension [n (%)]	518/3462 (15%)	375/727 (52%)	<0.001
Positive Surgical Margin [n (%)]	526/3462 (15%)	224/727 (31%)	<0.001
Robot-assisted RP [n (%)]	1223/3462 (35%)	244/727 (34%)	0.4
Nerve sparing RP [n (%)]	3198/3462 (92%)	597/727 (82%)	<0.001
OP duration, minutes [median, IQR]	80 (65–165)	83 (65–165)	0.1
Blood loss, ml [median, IQR]	200 (100–300)	200 (100–300)	<0.001
Postoperative radiation [n (%)]	102/1773 (6%)	83/391 (21%)	<0.001
Postoperative ADT [n (%)]	32/1774 (2%)	35/391 (9%)	<0.001
**Matched Cohort ^(a)^**			
	**No Upgrading**	**Upgrading**	***p***
No. of patients	656	656	
Age, years [median, IQR]	67 (63–72)	67 (62–72)	0.9
BMI, kg/m^2^ [median, IQR]	27 (25–29)	26 (25–29)	0.6
PSA preop., ng/mL [median, IQR]	6.9 (5.0–9.5)	8.0 (5.7–12)	<0.001
Gleason Score preOP [n (%)]			
6	318/656 (48%)	183/656 (28%)	<0.001
7a	340/656 (52%)	473/656 (72%)	
Gleason Score postOP [n (%)]			
6	211/656 (32%)		
7a	447/656 (68%)		
7b		477/656 (73%)	
8		131/656 (20%)	
9		47/656 (7%)	
10		1/656 (0.2%)	
Extracapsular extension [n (%)]	118/656 (18%)	338/656 (52%)	<0.001
Positive Surgical Margin [n (%)]	101/656 (15%)	197/656 (30%)	<0.001
Robot-assisted RP [n (%)]	240/656 (36%)	232/656 (35%)	0.7
Nerve sparing RP [n (%)]	541/656 (82%)	539/656 (82%)	1
OP duration, minutes [median, IQR]	90 (65–169)	85 (66–165)	0.9
Blood loss, ml [median, IQR]	200 (100–300)	200 (100–300)	0.1
Postoperative radiation [n (%)]	24/336 (7%)	79/375 (21%)	<0.001
Postoperative ADT [n (%)]	11/337 (3%)	33/375 (9%)	0.002

**Table 2:** Multivariable logistic regression analysis adjusted for postoperative upgrading as an independent risk factor for achieving good overall HRQOL (defined as a value of >70 of the Global Health Status domain of the EORTC-QLQ-C30 Questionnaire), continence recovery (defined as up to 1 Pad per 24 h) and satisfactory erectile function (defined as IIEF-5 ≥18). Adjustment for: “operative time”, “robot-assisted surgery”, “blood loss”, “nerve-sparing surgery”, “age”, and “BMI”. Bold values indicate *p*-values < 0.05 and were considered statistically significant. HRQOL: Health-related Quality of life; OR: Odds-ratio; 95% CI: 95% Confidence interval.

	Good Overall HRQOL			Continence Recovery			Satisfactory Erectile Function		
Risk Factor	OR	95% CI	*p*-Value	OR	95% CI	*p*-Value	OR	95% CI	*p*-Value
Postoperative upgrading	0.77	0.61, 0.97	**0.028**	0.82	0.63, 1.07	0.13	0.85	0.59, 1.21	0.4

## Data Availability

The data presented in this study are available on request from the corresponding author due to privacy concerns.
